# MicroRNA-34a promotes mitochondrial dysfunction-induced apoptosis in human lens epithelial cells by targeting Notch2

**DOI:** 10.18632/oncotarget.22597

**Published:** 2017-11-21

**Authors:** Fan Fan, Jianhui Zhuang, Peng Zhou, Xin Liu, Yi Luo

**Affiliations:** ^1^ Department of Ophthalmology, Eye & ENT Hospital, Fudan University, Shanghai, China; ^2^ Myopia Key Laboratory of The Health Ministry and Visual Impairment and Reconstruction Key Laboratory of Shanghai, Shanghai, China; ^3^ Department of Cardiology, Shanghai Tenth People's Hospital, Tongji University School of Medicine, Shanghai, China; ^4^ Parkway Health Hongqiao Medical Center, Shanghai, China

**Keywords:** miR-34a, HLECs, apoptosis, age-related cataract, Notch

## Abstract

**Purpose:**

Human lens epithelial cell (HLEC) apoptosis is a common pathogenic mechanism in age-related cataracts (ARC). While the function of microRNAs (miRNAs) in the eye is beginning to be explored using miRNA expression array, the role of miR-34a in regulating HLEC apoptosis remains unknown and requires further investigation.

**Methods:**

Quantitative reverse-transcript polymerase chain reaction (RT-PCR) was used to determine the expression level of miR-34a in cataractous and control samples. MiR-34a mimics and small interfering RNAs were transfected into SRA01/04. Cell apoptosis and oxidative stress were assessed by flow cytometry. The Dual-Luciferase Reporter Assay System was used to confirm whether miR-34a bound to the 3’-UTR of the target gene and blocked its activity. The potential roles of the identified target genes in apoptosis and mitochondria dysfunction were also evaluated.

**Results:**

The expression of miR-34a increased in lens epithelial samples of ARC compared with the transparent group (cataract 2.41±0.81 vs. control 1.20±0.44, P=0.005). In cultured SRA01/04, miR-34a increased reactive oxygen species production and induced apoptosis (early apoptosis: 45.55%±5.96% vs. 15.85%±4.93%, P<0.01; late apoptosis: 6.10%±2.67% vs. 0.95%±0.42%, P<0.01). Overexpression of miR-34a promoted mitochondria-mediated apoptosis through activation of caspase-9, disruption of the mitochondrial membrane potential, blocking of mitochondrial energy metabolism and enhancement of cytochrome C release. Furthermore, Notch1 and Notch2 were confirmed as putative targets of miR-34a, but only Notch2 was verified as the effector that triggered mitochondria-mediated apoptosis.

**Conclusion:**

MicroRNA-34a is increased in the cataractous lens and triggers mitochondria-mediated apoptosis and oxidative stress by suppressing Notch2.

## INTRODUCTION

Cataract formation represents a serious problem in the elderly and has a large impact on the healthcare budget. Aging and cataract formation are relatively complex phenomena. The pathogenesis of cataracts involves environmental exposures and genomic mutations that alter epigenetic patterning [1]. MicroRNAs represent one epigenetic mechanism underlying the regulation of gene expression.

Studies have provided evidence of the participation of miRNAs in multiple cellular functions, such as cell proliferation [2], cell apoptosis [3], stress response [4], and senescence [5]. The role of miRNAs in the eye is now being explored following detection using an miRNA expression array, but this area of research has not yet received much attention compared with miRNA studies on cancer cells, blood, and muscle tissue [6]. Previous reports have shown that miRNA expression profiles differ between the central epithelium of transparent and cataractous lens samples, suggesting that miRNAs that are differentially expressed may play roles in lenticular development and cataractogenesis. Of the 32 miRNAs identified as significantly differentially expressed between transparent and cataractous samples, microRNA-34a (miR-34a) exhibited the greatest decrease in expression in the transparent group [7]. MiR-34a is located in chromosomal region 1p36.23, which is involved in cell cycle arrest, apoptosis, differentiation and cellular development [8]. In glioma and medulloblastoma, miR-34a levels were lower than in normal brain tissue, which in turn directly bound to Notch1 and Notch2 3’-UTR and suppressed Notch1 and Notch2 expression [9]. However, little information is available regarding miR-34a in age-related cataracts (ARC). In this study, we explored the role of miR-34a in regulating apoptosis in human lens epithelial cells (HLECs).

## RESULTS

### miR-34a expression is increased in lens epithelial samples of ARC

**Figure 1:**
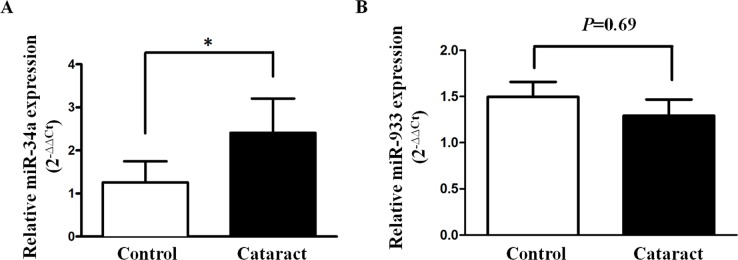
Expression status of miR-34a and miR-933 in age-related cataracts Comparison of the relative expression levels of miR-34a **(A)** and miR-933 **(B)** between control and age-related cataract groups.

Using a microarray array analysis, Wu et al. [7] identified 32 miRNAs with significantly different expression between transparent and cataractous samples. Among these differentially expressed miRNA, we selected miR-34a and miR-933 to confirm the results of the microarray study by quantitative RT-PCR. In the 30 ARC lens epithelial samples and 18 control samples, miR-34a exhibited significantly higher expression levels in the ARC compared with the control samples (cataract 2.41±0.81 vs. control 1.20±0.44, P=0.005, Figure 1A). MiR-933 also exhibited lower expression in the ARC samples, but the between-group difference in miR-933 expression did not achieve statistical significance (P=0.69, Figure 1B).

### miR-34a promotes apoptosis of HLECs via mitochondrial dysfunction

**Figure 2:**
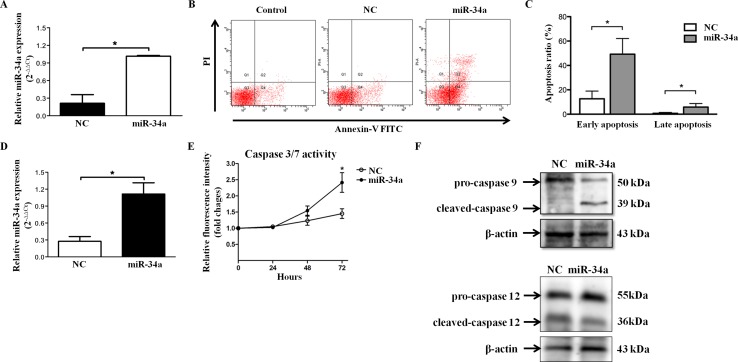
miR-34a induces apoptosis of HLECs **(A)** To evaluate the effect of miR-34a on SRA01/04 apoptosis, the efficiency of miR-34a mimics transfection was first confirmed by quantitative RT-PCR. **(B** and **C)** HLEC apoptosis was assessed 72 hours after transfection with scramble and miR-34a mimics by FACS analysis. Early apoptosis in the negative control group: 15.85% ± 4.93%; early apoptosis in the miR-34a overexpression group: 45.55% ± 5.96%; late apoptosis in the negative control group: 0.95% ± 0.42%; late apoptosis in the miR-34a overexpression group: 6.10% ± 2.67%. The apoptosis rate was significantly increased compared with control cells both in early and late apoptosis (P < 0.01). **(D)** To evaluate the effect of miR-34a on caspase-3 activity in HLECs, the efficiency of miR-34a mimics transfection was first confirmed by quantitative RT-PCR. **(E)** Caspase-3/7 activity was determined at the designated time points. Western blots showing caspase-9 and caspase-12 in HLECs after transfection with scramble and miR-34a mimics for 72 hours. β-actin served as an internal control. Data are representative of four separate experiments. ^*^ *P*<0.05 versus cells transfected with scramble mimics. NC=scramble mimics.

Next, we investigated HLEC function under conditions of miR-34a overexpression and explored the upstream and downstream regulatory mechanisms. Quantitative RT-PCR was performed to confirm the efficiency of transfection with miR-34a mimics in SRA01/04 (Figure 2A). We then investigated whether miR-34a played a functional role in HLEC apoptosis. Cell apoptosis was measured by Annexin-V and PI double staining, followed by flow cytometry. As shown in Figure 2B, miR-34a overexpression increased early and late apoptosis in HLECs. The apoptosis rate was significantly different from that of the control cells (early apoptosis: 45.55%±5.96% vs. 15.85%±4.93%, P<0.01; late apoptosis: 6.10%±2.67% vs. 0.95%±0.42%, P<0.01, Figure 2C). Caspase-3 is an executioner caspase. Its cleavage or activity is another marker of apoptosis [11]. Caspase-3/7 activity was detected 24 hours, 48 hours, and 72 hours after overexpressed transfection (Figure 2D). The caspase-3/7 activity gradually increased over time in both groups and reached a significantly higher level in miR-34a-overexpressing cells at 72 hours (miR-34a 0.19±0.02 vs. scramble 0.13±0.02, P=0.029, Figure 2E). These results suggest that miR-34a activates caspase-3/7 and triggers apoptosis in HLECs.

Caspase activation is central to apoptosis and can be initiated by any of three distinct mechanisms: ligand binding to death receptors, release of cytochrome C from mitochondria, or stress to the endoplasmic reticulum (ER) [12–14]. The intrinsic pathway via mitochondria involves caspase-9, whereas ER stress-mediated apoptosis activates caspase-12 [15, 16]. To confirm the upstream pathway, caspase-9 and caspase-12 were detected in SRA01/04 cells transfected with scramble and miR-34a mimics. As shown in Figure 2F, Western blot analysis revealed that cleaved-caspase-9 was significantly increased in miR-34a-transfected cells, whereas cleaved-caspase-12 presented no significant difference between the groups. These results indicate that mitochondrial dysfunction as an initial step provokes miR-34a-induced apoptosis via caspase-9 activation.

**Figure 3:**
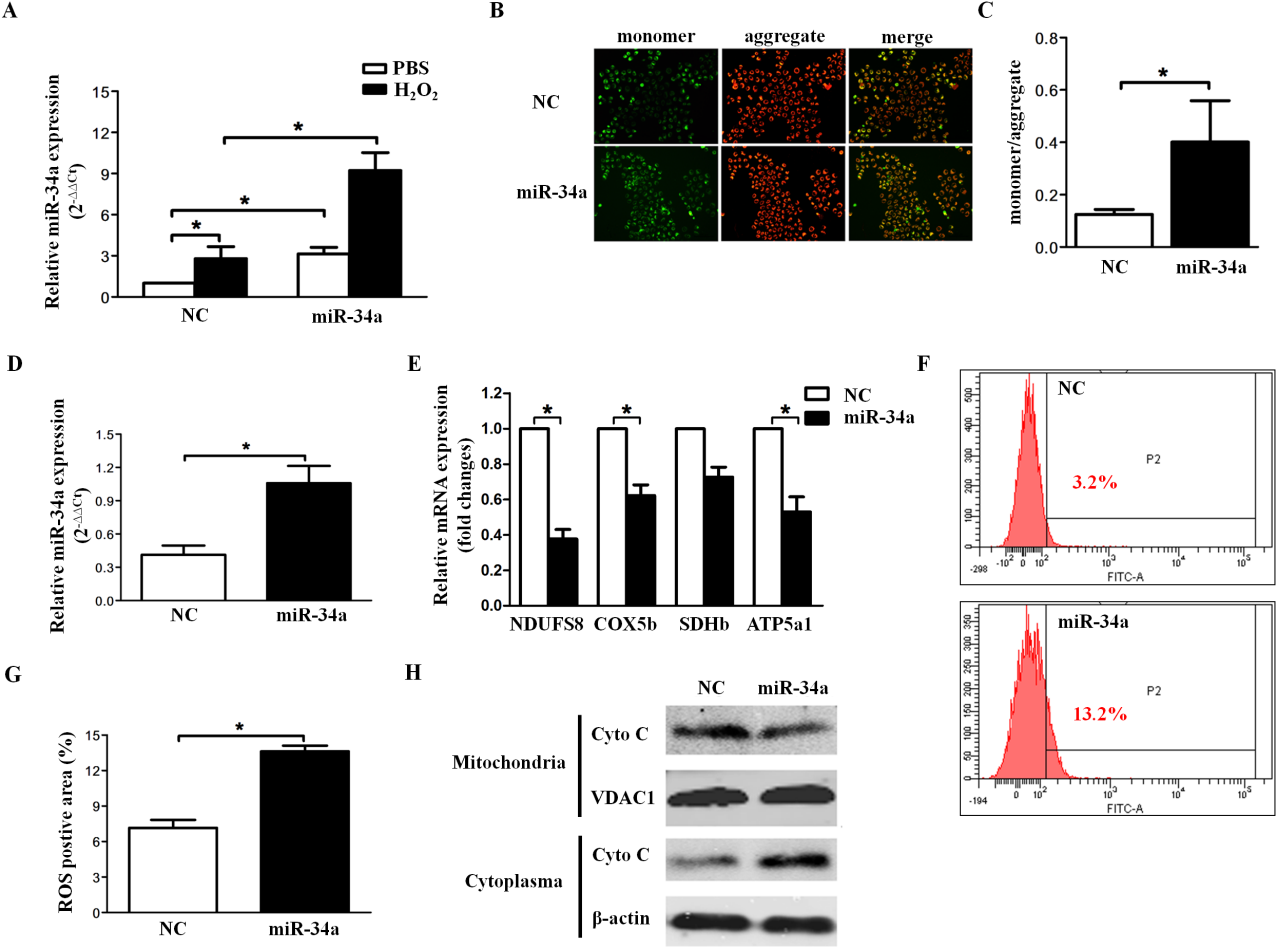
miR-34a disrupts mitochondrial biogenesis in HLECs **(A)** Human lens epithelial cells (SRA01/04) were cultured in serum-free medium for 24 hours, then transfected with scramble and miR-34a mimics for 72 hours, followed by H_2_O_2_ incubation for 1 hour. Quantitative RT-PCR indicated the expression levels of miR-34a in different groups. **(B)** Immunofluorescenceimages demonstrated the changes in the mitochondrial membrane potential of SRA01/04 after transfection with scramble and miR-34a mimics for 72 hours. **(C)** The fluorescence intensity was quantified in an automatic microplate reader (aggregates: excitation 525 nm, emission 590 nm; monomer: excitation 490 nm, emission 530 nm). **(D)** To evaluate the effect of miR-34a on the expression of the respiratory chain gene, the efficiency of miR-34a mimics transfection was first confirmed by quantitative RT-PCR. **(E)** Quantification of NDUFS8, COX5b, SDHb and ATP5a1 mRNA levels was performed by qRT-PCR. **(F** and **G)** Statistical results of the DCF positive area were obtained by flow cytometry analysis. **(H)** Western blots showed protein levels of cytochrome c in the cytoplasm and mitochondria. Data are the means ± S.E.M. of four experiments. ^*^ *P*<0.05 versus cells transfected with scramble mimics. NC=scramble mimics, ROS=reactive oxygen species.

Mitochondrial dysfunction includes ROS, energy metabolism and the mitochondrial apoptotic pathway. To evaluate the effect of miR-34a on MMP, the efficiency of miR-34a mimics transfection was first confirmed by quantitative RT-PCR (Figure 3A). Additionally, incremental miR-34a expression was observed in cells incubated with H_2_O_2_, which is known as one of the critical mediators of oxidative stress (Figure 3A). As shown in Figure 3B and 3C, the monomer/aggregates ratio was significantly higher in the presence of miR-34a, indicating that miR-34a overexpression leads to MMP disruption. The components of the mitochondrial electron transport chain include NDUFS8, SDHb, COX5b, and ATP5a1. [17] NDUFS8, SDHb, COX5b, and ATP5a1 mRNA were analyzed by quantitative RT-PCR 24 hours after transfection of SRA01/04 with scramble or miR-34a (Figure 3D). The results showed that the expression levels of three genes, NDUFS8, COX5b and ATP5a1, were significantly inhibited in the miR-34a-overexpressing group (P<0.01, Figure 3E), suggesting that the mitochondrial electron transport chain was blocked by enhanced miR-34a expression. Inhibition of the mitochondrial electron transport chain would lead to the subsequent formation of ROS. As presented in Figure 3F and 3G, DCF positive areas were significantly increased in miR-34a treated cell lines, confirming that miR-34a overexpression may promote ROS in HLECs.

Increasing evidence indicates that mitochondria-mediated apoptosis requires the release of cytochrome C from mitochondria and the activation of caspase-9 and caspase-3 to trigger a series of downstream apoptotic events [18]. Therefore, we examined the levels of cytochrome C protein in isolated mitochondrial and cytosolic fractions in control and miR-34a-overexpressing groups. Western blot analysis revealed that overexpression of miR-34a resulted in the translocation of cytochrome C from mitochondria to the cytoplasm (Figure 3H).

In summary, miR-34a overexpression causes mitochondrial outer membrane dysfunction, increasing its permeability and thus allowing the release of cytochrome C from mitochondria into the cytoplasm and the activation of caspase-3/7 and caspase-9 to trigger HLEC apoptosis.

### Notch1 and Notch2 are functional targets of miR-34a

**Figure 4:**
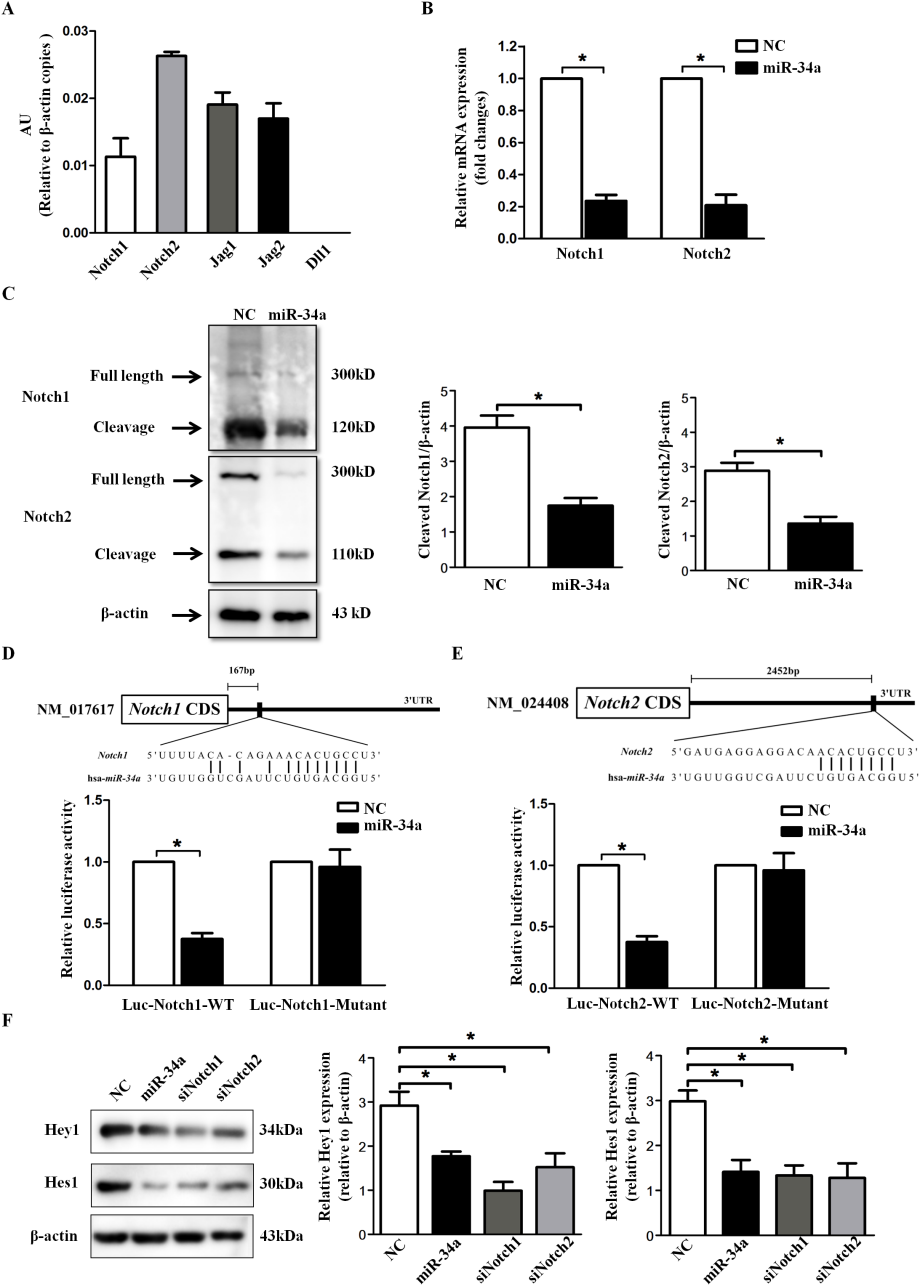
miR-34a directly targets Notch1 and Notch2 **(A)** The expression of five genes related to Notch signaling in human lens epithelial cell line SRA01/04 was quantified using qRT-PCR. **(B** and **C)** Analysis of Notch1 and Notch2 expression by qRT-PCR and Western blots after transfection with scramble and miR-34a mimics for 72 hours. **(D** and **E)** Construction of Luciferase-wildtype UTR vectors (Luc-Notch1-WT and Luc-Notch2-WT) and the corresponding luciferase-mutated UTR vectors (Luc-Notch1-Mutant and Luc-Notch2-Mutant). Transfection with miR-34a mimics significantly reduced luciferase activities, whereas it did not affect the luciferase activity in the mutant construct compared with scramble mimics transfection. **(F)** Western blots showed Hey1 and Hes1 in SRA01/04 after transfection with scramble mimics, miR-34a mimics, siNotch1 and siNotch2 for 72 hours. AU = absolute unit, NC=scramble mimics, WT=wild type. Data are the means ± S.E.M. of four experiments. ^*^ *P*<0.05 versus SRA01/04 transfected with scramble mimics.

The in silico miRNA target identification tools PicTar (http://pictar.mdcberlin.de/) and TargetScan 4.2 (http://www.targetscan.org/vert_42/) identified Notch1, Notch2, Dll1, Jag1 and Jag2 as potential targets of miR-34a. The expression levels of the five genes were evaluated in HLECs by absolute quantitative PCR. As presented in Figure 4A, among the five target genes, Notch2 exhibited the highest expression levels, while Dll1 was undetectable in HLECs (Figure 4A). Because Notch1 and Notch2 are both important receptors in the Notch signaling pathway, we hypothesize that Notch1 or Notch2 is a main target of miR-34a. RT-PCR and Western blot exhibited significant inhibition in Notch1 and Notch2 expression after miR-34a transfection in HLECs (Figure 4B and Figure 4C).

To confirm that Notch1 and Notch2 were putative targets of miR-34a, a Luciferase reporter construct carrying the 3’-UTR miR-34a potential binding site of Notch1 and Notch2 was constructed and co-transfected with either scramble or miR-34a mimics into HEK293T cells. As shown in Figure 4D and Figure 4E, compared with the control, transfection with miR-34a mimics significantly reduced luciferase activity (Notch1 wild type, P=0.035; Notch2 wild type, P=0.021). However, pre-miR scramble or pre-miR-34a did not affect the luciferase activity in the mutant construct, providing luciferase activities that were not significantly different from the activity of the control (Notch1 mutant, P=0.720; Notch2 mutant, P=0.814). These results demonstrate that miR-34a binds to Notch1 and Notch2 3’-UTR and influences their expression.

Furthermore, the downstream effectors of Notch signaling, Hey1 and Hes1, were examined to confirm that miR-34a could down-regulate the expression of Notch target genes. As shown in Figure F, similar to the results obtained for the siNotch1 and siNotch2 groups, we detected reduced expression of Hey1 and Hes1 after transfection with miR-34a mimics in SRA01/04 compared with the scramble siRNA group.

### Knockdown of Notch2 promotes cell apoptosis

**Figure 5:**
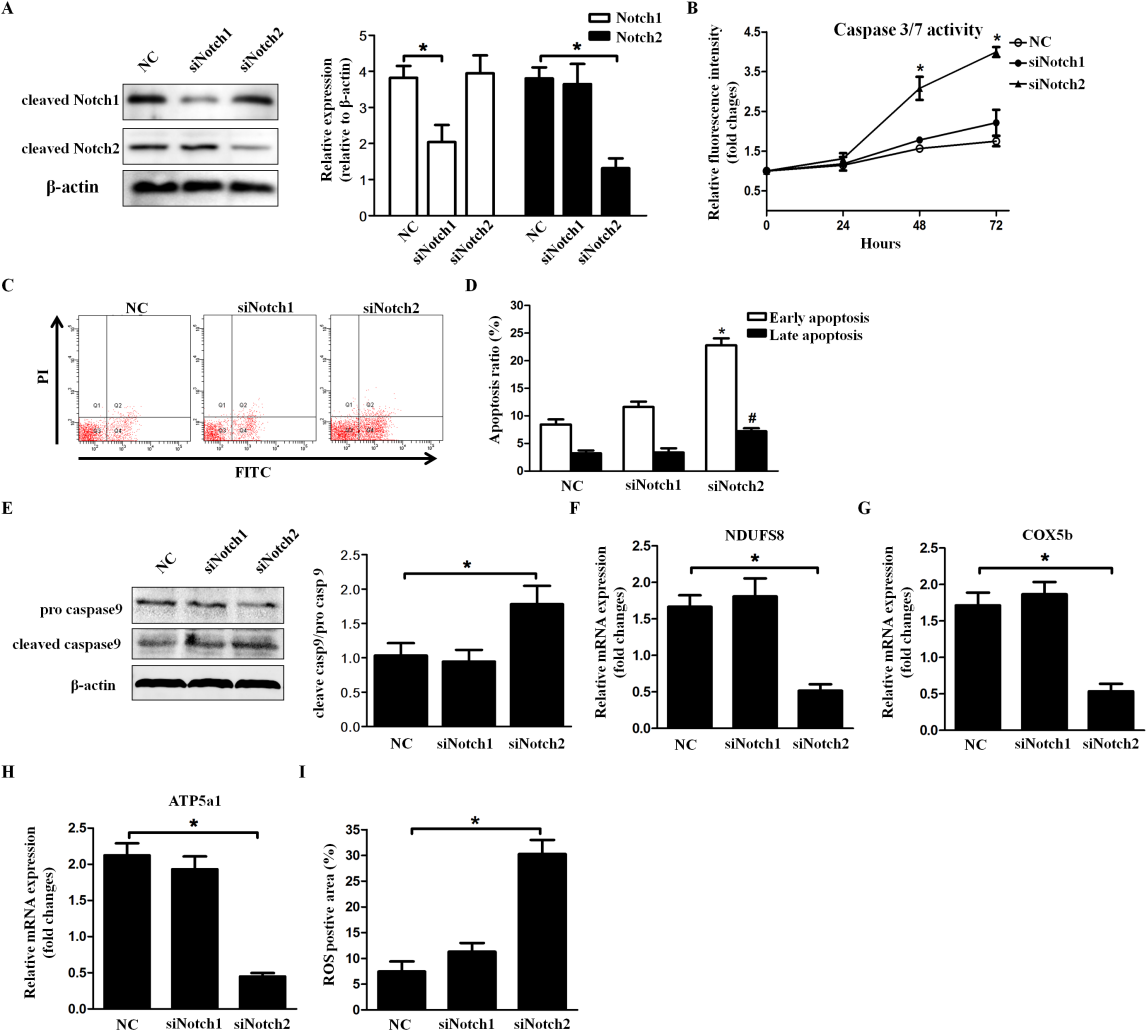
Knockdown of Notch2 aggravates HLEC apoptosis **(A)** The efficiency of Notch1/Notch2 knockdown in SRA01/04 was determined by Western blot analysis. **(B)** Caspase-3/7 activity at the indicated time was measured after transfection with Notch1/Notch2 siRNA. **(C** and **D)** Representative flow cytometry images and statistical results showed the early and late apoptosis ratio of SRA01/04 after transfection with Notch1/2 siRNA for 72 hours. **(E)** Western blots showed protein levels of pro- and cleaved-caspase-9 in SRA01/04 transfected with Notch1/2 siRNA. **(F-H)** Quantitative RT-PCR was performed to compare the expression levels of NDUFS8, COX5b and ATP5a1 among three groups. **(I)** Statistical results for the relative fluorescence intensity of DCF were obtained by flow cytometry analysis. Data are the means ± S.E.M. of five experiments. NC=scramble mimics. ^*^ *P*<0.05 versus cells transfected with scramble siRNA.

To determine whether Notch1 and Notch2 participate in apoptosis and mitochondrial function of HLECs, Notch1 and Notch2 were respectively knocked down by siRNA transfection into cells for 72 hours. The knockdown efficiency was assessed by Western blot analysis (Figure 5A). Caspase-3/7 activity increased significantly in the siNotch2-transfected group (P<0.05) compared with that in the si-Notch1-transfected group (Figure 5B). Similarly, in cells transfected with si-Notch2, early and late cell apoptosis was significantly increased compared with that of the negative control (early apoptosis: 22.31% ± 3.04% vs. 7.64% ± 2.15%, P=0.012; late apoptosis 6.83% ± 1.29% vs. 4.18% ± 0.97%, P=0.012), whereas there was no significant change in the cells transfected with si-Notch1 (P=0.276) (Figure 5C and 5D). In addition, we examined the expression of caspase-9, an apoptotic biomarker of the mitochondrial pathway. The Western blot results showed that pro-caspase-9 was alleviated and that cleaved-caspase-9 was augmented in si-Notch2-transfected cells, whereas no significant change was observed in si-Notch1-transfected cells (Figure 5E). We thoroughly analyzed the expression levels of respiratory chain genes NDUFS8, COX5b and ATP5a1 in the two groups by quantitative RT-PCR. The results indicated that only knockdown of Notch2 could reduce their expression (Figure 5F-5H). In other words, Notch2 impeded mitochondrial energy metabolism. In addition, the levels of ROS were significantly elevated in Notch2 knockdown cells (Figure 5I). In general, Notch2 induces HLEC apoptosis via mitochondrial dysfunction.

### Overexpression of N2-ICD reverses the effect of miR-34a on cell apoptosis

**Figure 6:**
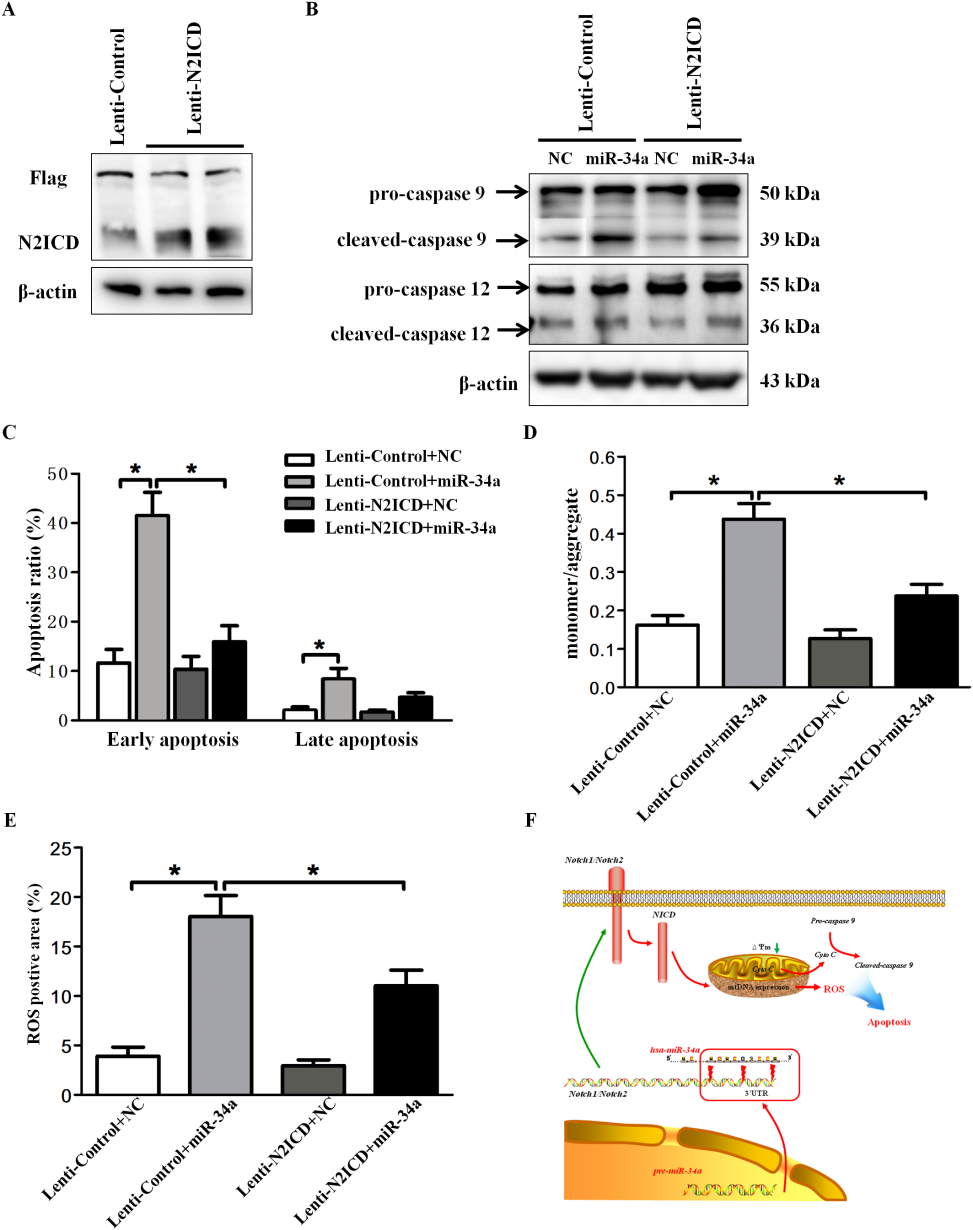
Overexpression of N2-ICD rescues HLEC apoptosis **(A)** Western blots were performed using specific antibodies (Flag-tag and Notch2) to evaluate Flag-tagged N2-ICD overexpression lentivirus expression levels in SRA01/04. **(B)** Following lentiviral transduction, SRA01/04 was transfected with scramble mimics and miR-34a mimics for 72 hours. Western blots showed caspase 9 and caspase 12 in SRA01/04. **(C)** Statistical results for the early and late apoptosis rate obtained by FACS analysis. **(D)** Following lentiviral transduction, SRA01/04 was transfected with scramble mimics and miR-34a mimics for 72 hours. The fluorescence intensity of mitochondrial membrane potential was quantified in an automatic microplate reader. **(E)** Statistical results for the relative fluorescence intensity of DCFH-DA were obtained by flow cytometry analysis. **(F)** Proposed schema of miR-34a mediated Notch pathway in the regulation of mitochondria dysfunction and apoptosis in HLECs. Mature miR-34a directly binds to Notch1 and Notch2 3’UTR, subsequently suppressing Notch1 and Notch2 transcription. Decreased Notch2 and N2-ICD result in mitochondria dysfunction and oxidative stress, leading to apoptosis of HLECs. Data are the mean ± S.E.M. of five experiments. Lenti=lentivirus, N2ICD=Notch2 Intracellular Domain, NC=scramble mimics. ^*^ P<0.05.

Because both miR-34a and Notch2 participated in HLEC apoptosis, we investigated whether Notch2 is required for miR-34a-induced apoptosis. As an initial step, we confirmed that transduction of pCMV-3Flag-U6-N2-ICD substantially stimulated N2-ICD expression in SRA01/04 cells relative to that in the control lentivirus group (Figure 6A). Co-transfection of pCMV-3Flag-U6-N2-ICD with miR-34a mimics in SRA01/04 cells significantly reversed the excessive cleavage of caspase-9 induced by miR-34a mimics but without changes in the expression of caspase-12 (Figure 6B). Consistent with the Western blot data, overexpression of N2-ICD mitigated miR-34a-mediated early apoptosis but did not significantly affect the late apoptosis of HLECs (Figure 6C). Given that miR-34a induced HLEC apoptosis through mitochondrial dysfunction, we further determined the rescue effect of N2-ICD on MMP and ROS in HLECs transfected with miR-34a. As shown in Figure 6D, overexpression of N2-ICD with lentiviral transduction markedly rescued the impaired MMP induced by miR-34a mimics. Likewise, overexpression of N2-ICD antagonized miR-34a-induced overproduction of ROS in SRA01/04 cells (Figure 6E).

Overall, we demonstrated that the expression of miR-34a leads to increased apoptosis and mitochondrial dysfunction, at least in part through Notch2 signaling, as illustrated in our sketch (Figure 6F).

## DISCUSSION

Cataracts, which are the most common cause of blindness worldwide, are significantly related to the aging process. Recent studies have proved that upregulation of miR-34a may activate apoptosis and cell cycle arrest [8]. For example, miR-34a is stimulated in the aging heart, and *in vivo* silencing or genetic deletion of miR-34a reduces age-associated cardiomyocyte cell death by inducing DNA damage responses and telomere attrition [19]. In addition, aging mesangial cells exhibit a significant upregulation of miR-34a, and overexpression of miR-34a facilitates the premature senescence of young mesangial cells [20]. Based on the microarray results of distinct expression profiles of miRNAs in the central epithelium of transparent and age-related cataractous human lenses [7], we showed that miR-34a is overexpressed in age-related cataractous lens epithelium. Similarly, in a case-control study, correlations among age, lens opacity, and miR-34a expression levels were evaluated in 110 patients, demonstrating positive correlations between high miR-34a levels and high lens opacity severity in cataracts [21].

Currently, many factors, such as diabetes mellitus, ultraviolet exposure, systemic drugs, and other ocular diseases, are known to be related to cataract formation [22, 23]. Among these disorders, oxidative stress with the subsequent formation of ROS has been proposed to be a major predisposing factor in ARC. Li et al. [24, 25] showed that lens epithelial cell apoptosis is a common cellular basis for the development of non-congenital cataract in human and mammalians. Therefore, we examined apoptotic biomarkers and cell functions in miR-34a-overexpressing HLECs to explore their role in cataractogenesis. Our results indicated that miR-34a activates HLEC apoptosis through mitochondrial dysfunction, including blocking electron transport, formation of ROS and attacking the mitochondrial membrane, further releasing cytochrome c into the cytoplasm and triggering caspase-9 activation (Figure 6F). This result is consistent with a previous study showing that overexpression of miR-34a induces the premature senescence of young mesangial cells by suppressing SOD2 and Txnrd2 together with a concomitant increase in ROS [20].

To identify putative target messenger RNAs of miR-34a, we used microRNA target prediction tools. Among the targets, Notch1, Notch2, Dlla, Jag1 and Jag2 are key components of the Notch signaling pathway, which is a highly conserved, cell-cell signaling pathway that regulates cell fate determination during development [26–28]. Notch1 and 2 are the receptors and Dll1, Jag1, and Jag2 are the ligands in the pathway. Notch receptors were found to be expressed in Drosophila and the developing rat eye as early as 1991 and 1997, respectively [29, 30]. *in vitro* studies of the differentiation of postnatal rat lens epithelial cells demonstrated that Notch2 signaling was activated during FGF-dependent secondary fiber cell differentiation, but no corresponding activation of Notch1 was detected [31]. The authors subsequently reported the lens phenotypes of Notch2 conditionally mutant mice, which exhibit severe microphthalmia, a disrupted fiber cell morphology, eventual loss of the anterior epithelium, fiber cell dysgenesis, denucleation defects, and cataracts [32]. Collectively, we propose that the five candidate genes of the Notch pathway may contribute to the development of ARC. First, we confirmed that Notch and Jag were expressed in HLECs. Because receptors play more important roles than ligands, we explored the relationship between miR-34a and Notch1/Notch2. The luciferase assay results confirmed that the 3’-UTR of Notch1 and Notch2 contains a target site for miR-34a binding. To further verify the key target of miR-34a, we next suppressed the expression of Notch1 and Notch2 by transfecting siRNAs into the cells and then assessing cell function. Our data showed that apoptosis and mitochondria dysfunction both occurred in Notch2-suppressed cells, whereas there was no significant change in Notch1-suppressed cells.

In summary, the age-induced expression of miR-34a and inhibition of its target, Notch2, regulate HLEC function during aging by inducing mitochondrial apoptosis. Therefore, Notch2 may be a crucial regulatory gene in cataractogenesis, warranting further investigation.

## MATERIALS AND METHODS

This study followed the tenets of the Declaration of Helsinki. The institutional review board of Fudan University Eye and Ear, Nose, and Throat Hospital approved our use of anterior capsule membranes from cataract eyes during surgery.

### Sample collection

Lens epithelium samples were obtained between July 2012 and December 2012. All patients underwent a complete preoperative ophthalmologic examination. Only those who had no ocular diseases other than age-related cataracts were included. Patients who had previously undergone ocular surgery or those with diabetes were excluded. The type and severity of cataracts were graded and recorded according to the modified version of the lens opacity classification system III (LOCSIII). Lenses with a score of CN4-6, C4-5 and P4-5 were selected as the age-related cataract group. Control lens epithelium samples with a LOCSIII score of C1, NC1, or P1 were obtained from the vitrectomy operation of epiretinal membranes. Lens epithelium samples were obtained by intact continuous curvilinear capsulorhexis, with care taken to avoid vascular contact or damage to the iris or other intraocular structures. Thirty eyes in the cataract group with ages ranging from 60 to 75 years old versus 18 eyes with ages ranging from 50 to 65 years old in the control group were collected. Each sample was prepared for RNA isolation and quantitative reverse-transcript polymerase chain reaction (RT-PCR). All samples were collected after obtaining informed consent from the patients.

### RNA isolation and quantitative RT-PCR

The expression levels of miR-34a were determined by quantitative RT-PCR. Briefly, the miRNeasy Mini Kit (Qiagen, Hilden, Germany) was used to isolate microRNAs from total RNA according to the manufacturer's instructions, and reverse transcription was performed using 1 μg of RNA with the PrimeScript RT reagent Kit (Takara, Tokyo, Japan). As an internal control, U6 was used for miRNA template normalization, and GADPH was used for other template normalizations. The amplification and detection of specific products was performed in triplicate using the SYBR Premix Ex Taq assay (Takara, Tokyo, Japan), and the relative expression levels between groups were calculated using the following equation: relative gene expression = 2^-(ΔCtsample-ΔCtcontrol)^. To assess the abundance of the Notch signaling pathway, β-actin is used as an internal reference to normalize the input DNA and to generate a standard curve generated from a standard DNA sample (Baomanbio, Shanghai, China). The amount of the gene of interest (Notch1, Notch2, Jag1, Jag2 and Dll1) was determined by the threshold cycle number (Ct) for each sample, relative to the standard curve. The percentage of gene copies of interest was calculated by dividing the GENE/β-actin ratio of a sample by the GENE/β-actin ratio of a standard sample. The primer sequences are listed in Supplementary Table 1.

### Cell culture and transfection

HLECs (SRA01/04 cell line) were purchased from the Tumor Center of the Chinese Academy of Medical Sciences, Beijing, China. They were grown in DMEM (Gibco, NY, USA) supplemented with 10% fetal bovine serum (Gibco, NY, USA) at 37°C with 5% CO_2_ in a humidified incubator. To examine the effect of miR-34a overexpression, scramble and miR-34a mimics (Sigma, St. Louis, MO, USA) were transfected into the cells using HiPerFect Reagent (Qiagen, Hilden, German) following the manufacturer's instruction. For functional analysis of Notch1 and Notch2, the SRA01/04 cells were transfected with scramble, Notch1 and Notch2 siRNA (Notch1 cat# SASI_Hs02_00350287; Notch2 cat# SASI_Hs01_00068800; Sigma, St. Louis, MO, USA) using the same method to inhibit expression. The down-regulation of miR-34a, Notch1 and Notch2 was confirmed by quantitative RT-PCR and Western blot analysis.

### Transduction of HLECs with lentivirus to upregulate Notch2 intracellular domain

The construction of vectors carrying Notch2 Intracellular Domain (N2-ICD) and the packaging of lentivirus were performed by the GENECHEM Corporation (Shanghai, China). The lentivirus (pCMV-3Flag-U6-N2-ICD) was stored at -80°C and prepared to infect HLECs. SRA01/04 was seeded into 6-well plates at approximately 70-80% confluence. Fresh medium without antibiotics was added, followed with supplementation of diluted lentivirus solution. The following experiments were performed 48 h after transfection.

### Detection of apoptosis

Cellular apoptosis was determined with the Annexin V-FITC/PI Apoptosis Detection kit (eBioscience, San Diego, CA, USA) using flow cytometry as described previously [10]. Briefly, SRA01/04 cells were centrifuged, washed with ice-cold PBS, and resuspended in 250 μl of binding buffer. The cells were then incubated with Annexin V-FITC and PI in the dark at room temperature for 15 min and subsequently analyzed by flow cytometry (FACSCalibur, BD Biosciences, San Jose, USA). Cells displaying only Annexin V-positive staining were considered to be in early apoptosis, whereas cells stained with Annexin V and PI were considered to be in late apoptosis.

### Measurement of caspase-3/7 activity

Apoptosis was evaluated using the Caspase-Glo(R) 3/7 Chemiluminescent Assay kit (Promega, USA) following the manufacturer's protocol. SRA01/04 cells were seeded in 12-well plates at a density of 5 × 10^4^ cells/well. After 24 hours, the cells were transfected with scramble and miR-34a mimics. After incubation, the cells were trypsinized, washed with PBS, and processed. The caspase assay was performed in 96-well white-walled plates by adding 100 μl of Caspase-Glo(R) 3/7 reagent to each well containing 1 × 10^4^ and 2 × 10^4^ cells in 100 μl of culture medium. After a one-hour incubation in the dark at room temperature, the luminescence (index of caspase-3/7 activity) was measured using a VersaDoc MP System equipped with Quantity One(R) version 4.6 software (Bio-Rad). Luminescence values from the blank reaction (vehicle-treated cells) were subtracted from the experimental values.

### Western blot analysis

Cells were lysed in RIPA lysis buffer and phenylmethanesulphonyl fluoride at 4°C for 30 min. Mitochondria were isolated using the Mitochondria Isolation Kit for Cultured Cells (Thermo Scientific, Rockford, IL, USA) and prepared for Western blot analysis. A total of 30 μg of protein was separated in a 6% to 12.5% SDS-PAGE gel, transferred to a PVDF membrane and probed with the following antibodies overnight at 4°C: caspase-9 (cat# ab202068, 1:600 dilution, Epitomics), cytochrome C (cat# ab133504, 1:400 dilution, Epitomics), caspase-12 (cat# 3282-100, 1:1000 dilution, BioVision), VDAC1 (sc-390996, 1:600 dilution, Santa Cruz), Notch1 (cat# 3282-100, 1:400 dilution, Santa Cruz), Notch2 (cat# 5732, 1:1000 dilution, Cell Signaling Technology), Hes1 (cat# ab71559, 1:400 dilution, Abcam) and Hey1 (cat# ab22614, 1:400 dilution, Abcam). After incubation with either HRP-conjugated anti-rabbit or anti-mouse antibodies, immunoreactive bands were visualized by chemiluminescence (ECL Plus; GE Health Care, Piscataway, NJ), and the membranes were exposed to light film (BioMax MR; Kodak, Rochester, NY). The β-actin antibody (1:2000 dilution; Santa Cruz) was used as a loading control.

### Mitochondrial membrane potential assay

The mitochondrial membrane potential (MMP) was analyzed using a JC-1 fluorescence probe kit (Beyotime, China). The lipophilic and cationic fluorescent dye JC-1 (5,5’,6,6’-tetrachloro-1,1’,3,3’-tetraethylbenzimidazolocarbocyanine iodide) is capable of selectively entering mitochondria, where it forms aggregates and emits red fluorescence when MMP is high. At low MMP, JC-1 cannot enter mitochondria and forms monomers that emit green fluorescence. The ratio of green to red fluorescence provides an estimate of the changes in MMP. The monomer/aggregates ratio was quantified by an automatic microplate reader (aggregates: excitation 525 nm, emission 590 nm; monomer: excitation 490 nm, emission 530 nm). Transfected cells seeded in a 6-well plate were treated with JC-1 (5 μg/ml) at 37°C for 1 hour. After washing with serum-free medium, the fluorescence intensity was immediately measured using fluorescence microscopy (Leica, Germany).

### Detection of reactive oxygen species (ROS)

ROS production was quantified using the 2’,7’-dichlorofluorescin-diacetate (DCFH-DA, Sigma, St. Louis, MO, USA) method based on the ROS-dependent oxidation of DCFH-DA to DCF. Cells were incubated with 10.0 μM DCFH-DA for 30 min at 37°C for 5 min. The fluorescence intensity and positive area of ROS were determined by flow cytometry.

### Dual-Luciferase analysis

HEK293T cells were maintained in DMEM supplemented with 10% fetal bovine serum. A construct in which a fragment of the 3’-UTR of Notch1/Notch2 mRNA contains the putative miR-34a binding sequence was cloned into a psiCHECK2 luciferase reporter vector (Promega, Madison, WI, USA). Constructs carrying the mutated fragment of the 3’-UTR of Notch1 and Notch2 mRNA without the putative miR-34a binding sequences were used as the mutated controls. To determine whether miR-34 could bind to the Notch-1 or Notch-2 3’-UTR, HEK293T cells were seeded in 24-well plates and co-transfected using FuGENE 6 (Promega, Madison, WI, USA) with 10 nM miR-34a mimic or scramble miRNA mimic and 2.5 μg psiCHECK2 vector containing mutant or putative fragments of the 3’-UTR of Notch1/Notch2. The oligonucleotide fragments of mutant Notch1-3’-UTR and Notch2-3’-UTR inserted into the luciferase vector were assembled using chemical synthesis. These regions did not contain miR-34a consensus-binding element ACACTGCC. The amplicons were inserted into vectors downstream of the cDNA for the firefly luciferase reporter gene. Sequences of mutant luciferase reporter are presented in Supplementary Table 2. After 48 hours of transfection, the relative luciferase expression normalized to *Renilla* activity was measured using an automatic micro-plate reader with the Dual-Luciferase Reporter Assay System (Promega, Madison, WI, USA).

### Statistical analysis

Each experiment was repeated at least three times independently. Statistical analyses were performed with SPSS 14.0 (SPSS Inc., Chicago, IL, USA). All values are reported as means ± S.E.M. Differences between groups were analyzed using Student's t-test. One-way ANOVA was used to compare multiple groups, when appropriate, with Bonferroni correction for the post hoc analysis. *P*<0.05 was considered statistically significant.

## CONCLUSION

Together, these results identify the age-induced expression of miR-34a and inhibition of its target, Notch2, as key mechanisms underlying the regulation of HLEC function during aging through the induction of mitochondria-mediated apoptosis.

## SUPPLEMENTARY MATERIALS TABLES



## Abbreviations

ARC: age-related cataracts
HLECs: human lens epithelial cells
