# Supplementary material for: MicroRNA-34a promotes mitochondrial dysfunction-induced apoptosis in human lens epithelial cells by targeting Notch2
Source: Oncotarget. 2017 Nov 21;8(66):110209–20. doi: 10.18632/oncotarget.22597 (PMC5746377; doi:10.18632/oncotarget.22597)
Supplement: Supplementary file 1 [file oncotarget-08-110209-s001.pdf]

MicroRNA-34a promotes mitochondrial dysfunction-induced apoptosis in human lens epithelial cells by targeting Notch2

SUPPLEMENTARY MATERIALS

Supplementary Table 1: Primers for Quantitative RT-PCR

Gene	Primers (5'→3')
miR-34a	F: CGG CGT GGC AGT GTC TTA G
	R: GTC GTA TCC AGT GCA GGG TCC GAG GTA TTC GCA CTG GAT ACG ACA CAA CC
U6	F: CTC GCT TCG GCA GCA CAT A
	R: GTC GTA TCC AGT GCA GGG TCC GAG GTA TTC GCA CTG GAT ACG ACA AAA TA
Notch1	F: TGG ACC AGA TTG GGG AGT TC
	R: GCA CAC TCG TCT GTG TTG AC
Notch2	F: CCT TCC ACT GTG AGT GTC TGA
	R: AGG TAG CAT CAT TCT GGC AGG
Jagged1	F: GTC CAT GCA GAA CGT GAA CG
	R: GCG GGA CTG ATA CTC CTT GA
Jagged2	F: TGG GAC TGG GAC AAC GAT AC
	R: AGT GGC GCT GTA GTA GTT CTC
Dll1	F: TGT GAC GAG TGT ATC CGC TAT
	R: GTG TGC AGT AGT TCA GGT CCT
NDUFS8	F: CCA TCA ACT ACC CGT TCG AG
	R: TAG ATG CAC TTG GTC ATG TCG
COX5b	F: TGT GAA GAG GAC AAT ACC AGC G
	R: CCA GCT TGT AAT GGG CTC CAC
SDHb	F: GAC ACC AAC CTC AAT AAG GTC TC
	R: GCT CAA TGG ATT TGT ACT GTG C
ATP5a1	F: ATG ACG ACT TAT CCA AAC AGG C
	R: CGG GAG TGT AGG TAG AAC ACA T
GADPH	F: GGG TGT GAA CCA CGA GAA AT
	R: ACT GTG GTC ATG AGC CCT TC

F: Forward primer; R: Reverse primer.

Supplementary Table 2: Sequence of mutant luciferase reporter

Luc-Notch1-Mutant	TCTAGAGGGCCGACCAGAGGAGCCTTTTTAAAACAC<br>ATGTTTTTATACAAAATAAGAACGAGGATTTTAATTT<br>TTTTTAGTATTTATTTATGTACTTTATCTATTCTGAGA<br>AAACAAGCAAGTTCTGAGAGCCAGGGTTTTCC<br>TACGTAGGATGTCTAGA
Luc-Notch1-Mutant	TTTCTAGAGTTTCTTTTTCTTGGACTACTTTAATTTGGA<br>TCCTTTGGGTTTGGAGAAAGGGAATGTGAAAGCTGTC<br>ATTTTTCAAACTTTTTACTGATCTCTTAGATTTTAAGAA<br>CTCTTGAATTGTGTGGTATCTAATAAAAGGGAAGGTAA<br>GATGGATAATCACTTTCTCATTTGGGTTCTGTCTAGACC
